# Comparative efficacy and safety of JAK inhibitors as monotherapy and in combination with methotrexate in patients with active rheumatoid arthritis: A systematic review and meta-analysis

**DOI:** 10.3389/fimmu.2022.977265

**Published:** 2022-09-29

**Authors:** Li Liu, Yi-Dan Yan, Fang-Hong Shi, Hou-Wen Lin, Zhi-Chun Gu, Jia Li

**Affiliations:** ^1^ Department of Pharmacy, Ren Ji Hospital, Shanghai Jiao Tong University School of Medicine, Shanghai, China; ^2^ School of Medicine, Tongji University, Shanghai, China; ^3^ Department of Rheumatology, Ren Ji Hospital, Shanghai Jiao Tong University School of Medicine, Shanghai, China

**Keywords:** janus kinase inhibitors, monotherapy, combination therapy, methotrexate, rheumatoid arthritis

## Abstract

**Background:**

We aim to evaluate the efficacy and tolerability of Janus kinase inhibitors (JAKi) as monotherapy and in combination with methotrexate (MTX) in active rheumatoid arthritis (RA).

**Methods:**

Medline, EMBASE, and Cochrane Library were systematically searched to identify relevant randomized controlled trials (RCTs). Pooled analysis was conducted using random-effects model, along with the risk difference (RD) and 95% confidence intervals (CIs).

**Results:**

Three RCTs, including 2,290 patients, were included. JAKi (tofacitinib, baricitinib, and filgotinib) plus MTX displayed a higher proportion of patients meeting the American College of Rheumatology (ACR) criteria than JAKi alone at week 52 (ACR20 RD 0.032; 95% CI −0.027 to 0.091; ACR50 RD 0.050; 95% CI 0.003 to 0.097; ACR70 RD 0.056; 95% CI 0.012 to 0.100). Similar results were observed for ACR20/50/70 at week 24. No significant difference was found between two regimens for the proportion of patients achieving Health Assessment Questionnaire disability index (HAQ-DI) improvement ≥ 0.22 at weeks 24 and 52. Regarding low disease activity and remission achievement, JAKi in combination with MTX, contributed higher response rates than JAKi alone at weeks 24 and 52. Compared with JAKi monotherapy, combination therapy had a higher risks of treatment-emergent adverse events (TEAEs) and adverse events (AEs) leading to study discontinuation.

**Conclusion:**

JAKi combined with MTX demonstrated superiority to JAKi monotherapy in terms of ACR responses, low disease activity and remission achievement. The two regimens presented comparable physical functioning measured by HAQ-DI improvement and similar tolerability, except for high risks of TEAEs and AEs leading to study discontinuation in combination therapy.

**Systematic Review Registration:**

https://www.crd.york.ac.uk/PROSPERO/, identifier CRD42021288907.

## Introduction

Rheumatoid arthritis (RA) is a systemic autoimmune disease characterized by painful, swollen joints and progressive bone erosion that affects physical functioning and quality of life (1). The therapeutic landscape of RA has been rapidly changing in recent years. Besides conventional synthetic disease-modifying antirheumatic drugs (csDMARDs) including methotrexate (MTX) and biologic DMARDs (bDMARDs), a new class of targeted synthetic DMARDs (tsDMARDs), represented by Janus kinase inhibitors (JAKi), has been introduced in the clinical practice for RA treatment. The American College of Rheumatology (ACR) and European League Against Rheumatism (EULAR) recommends starting treatment for RA with csDMARDs, preferably MTX (2, 3). JAKi could be added to csDMARDs if the treatment target is not achieved with the initial strategy, but is complicated by poor prognostic factors (2). Pivotal randomized controlled trials (RCTs) have proven the favorable efficacy and safety of JAKi combined with csDMARDs in RA, especially in combination with MTX (4–9).

The Janus kinase signal transducer and activator of transcription (JAK/STAT) pathway, which is implicated in the pathogenesis of RA, involves four members: JAK1, JAK2, JAK3, and TYK2 (10, 11). JAKi, as small molecules, inhibit the JAK/STAT pathway and block intracellular signaling mediated by multitudinous proinflammatory cytokines and other molecules, contributing to RA improvement (12). Currently, there are several licensed JAKi for RA treatment. Tofacitinib, a first-generation JAKi with predominant JAK1/JAK3 selectivity, was approved for RA treatment by the Food and Drug Administration in 2012 (13). Other licensed JAKi include baricitinib, which selectively inhibits JAK1/JAK2, and upadacitinib and filgotinib, which selectively inhibit JAK1 (14). JAKi are generally tolerated well with an acceptable safety profile; however, there are some specific safety concerns, such as herpes zoster infection, malignancy, and venous thromboembolism (12, 15).

Although established recommendations suggest dual therapy (JAKi plus MTX or other csDMARDs), patients under JAKi monotherapy have been estimated to account for approximately one-third in real-world practice due to intolerance of, or noncompliance, with MTX (16, 17). Published articles on tofacitinib indicated that based on clinical experience, JAKi monotherapy was suitable for approximately 60–70% of patients with RA and offered several practical advantages like reduction in medical expense, reduced csDMARDs-related adverse events and convenience of use (18). Previous RCTs, namely ORAL Solo (19), ORAL Start (20), RA-BEGIN (21), and SELECT-MONOTHERAPY (22), also validated the efficacy of JAKi monotherapy in RA treatment, with acceptable tolerability.

Currently, there is controversy over whether JAKi monotherapy has comparable efficacy and safety to JAKi plus MTX. However, comparisons of the two therapeutic regimens for the treatment of RA are rare. A phase 3b/4, double-blind, head-to-head RCT (ORAL Strategy) assessed the comparative efficacy of tofacitinib as a monotherapy or with MTX. The results were defined as statistically inconclusive because tofacitinib alone did not show non-inferiority to tofacitinib and MTX in the ACR50 response rate at six months (23). In contrast, the RA-BEGIN trial indicated that the efficacy of baricitinib monotherapy (ACR20 response rate at week 24) was similar to that of the combination with MTX; however, the study design lacked statistical comparisons between the concerned arms (21). As a result, we performed a meta-analysis in light of available data from recent RCTs to compare the efficacy and tolerability of JAKi as monotherapy and as combination therapy with MTX in the treatment of patients with active RA. We present the following article in accordance with the PRISMA reporting checklist.

## Methods

This meta-analysis was conducted according to the Preferred Reporting Items for Systematic Review and Meta-Analysis statement (24), and a prior protocol for this study was registered at the International Prospective Register of Systematic Reviews (PROSPERO: CRD42021288907 URL: https://www.crd.york.ac.uk/PROSPERO/#recordDetails).

### Literature search

We systematically searched Medline, EMBASE, and Cochrane Library to identify potentially eligible studies until May 17, 2022. Details of the study search process are presented in **Supplementary Table 1**. ClinicalTrials.gov was also searched to identify unpublished trials. The search was restricted to RCTs, human participants, and English publications. Cited references, meta-analyses, and reviews were reviewed to identify additional studies.

### Study selection

To determine study eligibility, two reviewers (LL and Y-DY) independently screened all titles and abstracts, and all papers were assessed based on the entry criteria. Any disagreements were resolved through discussion with the corresponding authors (JL, Z-CG and H-WL). Studies meeting the following criteria were included: (1) inclusion of patients with RA according to a standardized diagnostic classification system (ACR 1987 or EULAR/ACR 2010 criteria) (25, 26); (2) original reports of phase II and phase III RCTs; (3) JAKi therapy as monotherapy and in combination with MTX; and (4) available data on the efficacy and safety endpoints. The exclusion criteria for studies were: conference abstracts, reviews, letters, editorials, case reports, observation studies, long-term extension studies and *post hoc* analyses.

### Outcomes of interest

To ensure that sufficient data were used in the meta-analysis and to reduce the influence of confounding factors, the primary efficacy outcomes were regarded as the proportions at week 52 of patients achieving an ACR20/50/70 response, which means 20%/50%/70% improvement, respectively, in the ACR criteria (27); the proportion of patients sustaining low disease activity (as defined by Disease Activity Score in 28 joints, erythrocyte sedimentation rate or C-reactive protein [DAS28-4 (ESR/CRP)] ≤ 3.2, Simplified Disease Activity Index [SDAI] ≤ 11, Clinical Disease Activity Index [CDAI] ≤ 10); the proportion of patients attaining remission (as defined by DAS28-4 [ESR/CRP] < 2.6, SDAI ≤ 3.3, CDAI ≤ 2.8) at week 52; and the proportion of patients with Health Assessment Questionnaire disability index (HAQ-DI) improvement ≥ 0.22 at week 52. Secondary efficacy outcomes included the measures obtained at week 24. The safety outcomes were incidence at week 52 of treatment-emergent adverse events (TEAEs), serious adverse events (SAEs), adverse events (AEs) leading to study discontinuation, deaths, serious infections, herpes zoster infection, opportunistic infections, malignancy, venous thromboembolism (VTE), and major adverse cardiovascular events (MACE).

### Data extraction and quality assessment

Data were extracted into a pre-specified electronic form, including study characteristics (study name, NCT number, publication time, and duration of interventions), patient demographics (mean age, duration of RA, disease activity status), and reported outcomes of interest. Two independent reviewers (LL and Y-DY) performed the methodological quality assessment using the Cochrane Collaboration Risk of Bias Tool (28). Any dispute was resolved by consensus or consultation with the corresponding authors (JL, Z-CG and H-WL).

### Statistical analyses

All statistical analyses were performed using Stata version 13.1 (Stata Corporation, College Station, Texas, USA). Meta-analysis estimates of the studies were derived and presented as forest plots. Risk difference (RD) and 95% confidence intervals (CIs) were calculated by applying a Mantel-Haenszel random-effects model. An RD more than 0 indicated a higher trend in JAKi combination therapy than in monotherapy. The *I²* test was used to test the heterogeneity among the studies (> 50% considered significant heterogeneity) (29). Statistical significance was set at *P* < 0.05.

## Results

### Study selection and characteristics

**Figure 1:**
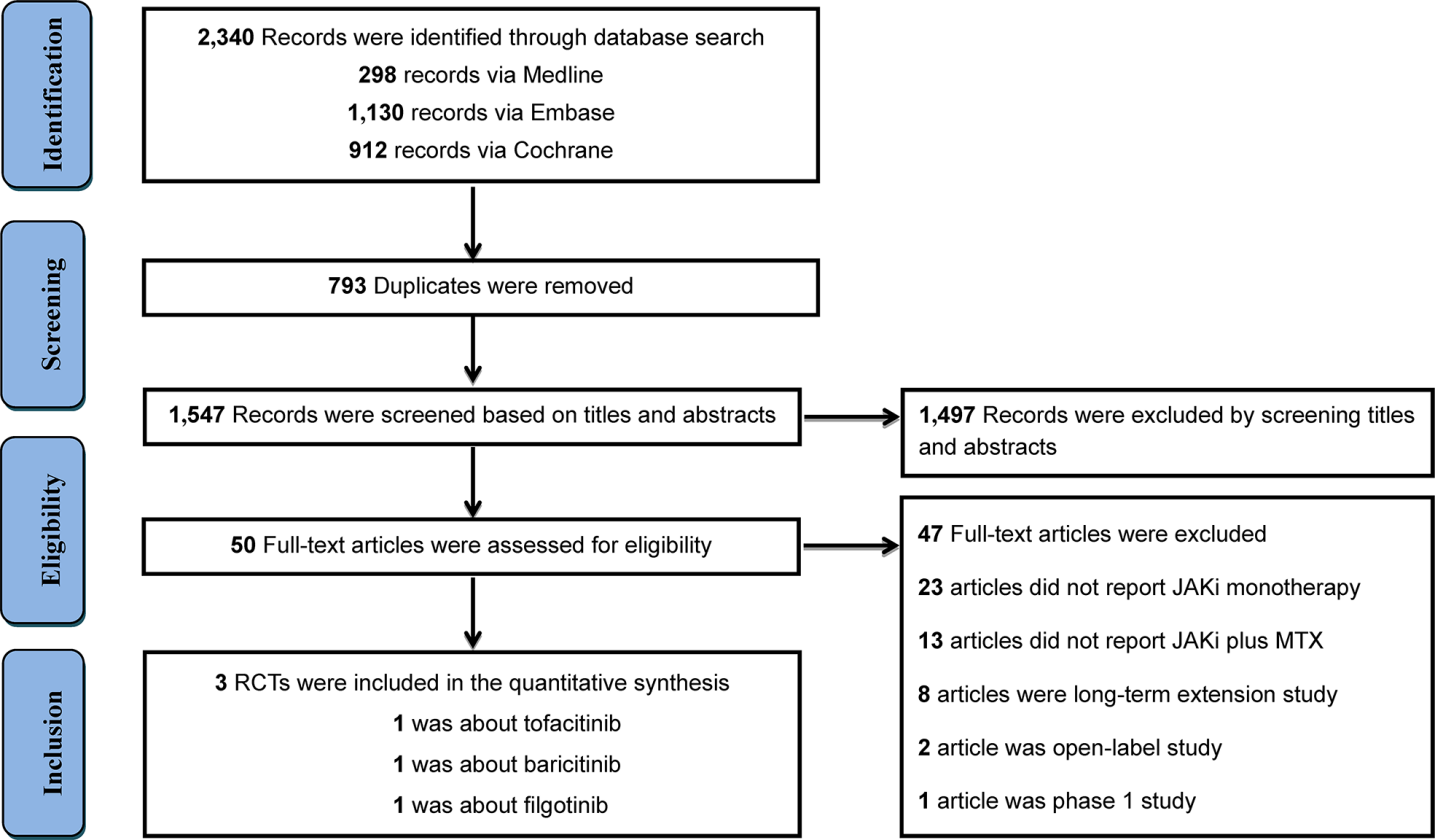
PRISMA diagram of the selection of eligible randomized controlled trials.

Among 2,340 records revealed by the literature search, 50 articles and abstracts were of potential interest. We excluded 47 articles after checking the full text. Detailed reasons were displayed in **Supplementary Table 2**. Further investigation resulted in three RCTs comprising 2,290 RA patients meeting the inclusion criteria (21, 23, 30), with 1,007 patients in the JAKi plus MTX combination arm and 753 patients in the JAKi monotherapy arm (**Figure 1**). All studies were registered on clinicaltrials. gov and we obtained the complete results. Among the included trials, one trial employed tofacitinib, one trial employed baricitinib, and the remaining one used filgotinib. **Table 1** presents the characteristics of the included studies. Patients with RA who had no or limited prior exposure to MTX and who had an inadequate response to MTX were included. The study duration was 52 weeks (one year for one study). MTX was administered orally once weekly, and dose of MTX ranging from 10 mg to 25 mg. The mean age ranged from 50.1 years to 53.2 years. The average disease duration of RA was 3.1 years in the JAKi combination group and 4.2 years in the JAKi monotherapy group. The mean SDAI and CDAI were 41.8, 39.7 in the combination group and 41.2, 39.3 in the JAKi monotherapy group, respectively.

**Table 1:** Characteristics of included randomized controlled trials.

Study	ORAL Strategy^23^		RA-BEGIN^21^		FINCH 3^30^	
Study year	2017		2017		2021	
NCT number	NCT02187055		NCT01711359		NCT02886728	
Study duration	1 year		52 weeks		52 weeks	
Mean age (years)	50.1		50.3		53.2	
Phase of study	IIIb/IV		III		III	
Study location	worldwide		worldwide		worldwide	
Included arms	Tofa 5 mg	Tofa 5 mg + MTX^a^	Bari 4 mg	Bari 4 mg + MTX^b^	Filg 200 mg	Filg 200 mg + MTX^c^
No. of patients	384	376	159	215	210	416
Duration of disease (years)	6.1	5.4	1.9	1.3	2.6	1.9
DAS28-4 (ESR)	6.5	6.6	6.6	6.6	N	N
DAS28-4 (CRP)	5.7	5.8	5.9	5.9	5.8	5.7
SDAI	40.2	41.6	43	43	41.8	41.3
CDAI	38.6	39.7	40	40	40.0	39.5
HAQ-DI	1.6	1.6	1.6	1.6	1.6	1.5
Race (%)						
White	77	76	N	N	64	67
Black or African American	3	5	N	N	4	4
Asian	11	10	N	N	22	22
Others	9	9	N	N	10	7

Data are mean; Tofa, Tofacitinib; Bari, Baricitinib; Filg, Filgotinib; No., number; N, unknown.

^a^MTX was administered orally once weekly, starting with 10 mg/week and escalating to 15 mg at week 4 and 20 mg at week 8.

^b^MTX was initiated at 10 mg/week and, if tolerated, increased to 20 mg/week by week 8.

^c^MTX was administrated with a stable dose of 15-25 mg per week.

### Risk of bias

**Table 2** presents the quality assessment results of included RCTs. The included RCTs satisfied four tool items (random sequence generation, blinding of participants and personnel, incomplete outcome data, and selective reporting). One study did not satisfy the blinding of outcome assessment item and was judged as moderate risk bias. Regarding allocation concealment, one study did not present the allocation concealment method and was evaluated as having an unclear risk bias. Overall, one study was judged to have a moderate risk of bias, and the rest were considered to have a low risk of bias.

**Table 2:** Quality assessment results of included randomized controlled trials.

Study	ORAL Strategy ^23^	RA-BEGIN ^21^	FINCH 3 ^30^
Random sequence generation	L	L	L
Allocation concealment	L	U	L
Blinding of participants and personnel	L	L	L
Blinding of outcome assessment	M	L	L
Incomplete outcome data	L	L	L
Selective reporting	L	L	L

L, low risk; M, moderate risk; U, unclear risk.

One study did not satisfy the item of blinding of outcome assessment and was judged as moderate risk bias. With regards to allocation concealment, one study did not present the allocation concealment method and was evaluated as unclear risk bias. Overall, one study was judged to have a moderate risk of bias and the remaining two were considered to have a low risk of bias.

### Primary efficacy outcomes

**Figure 2:**
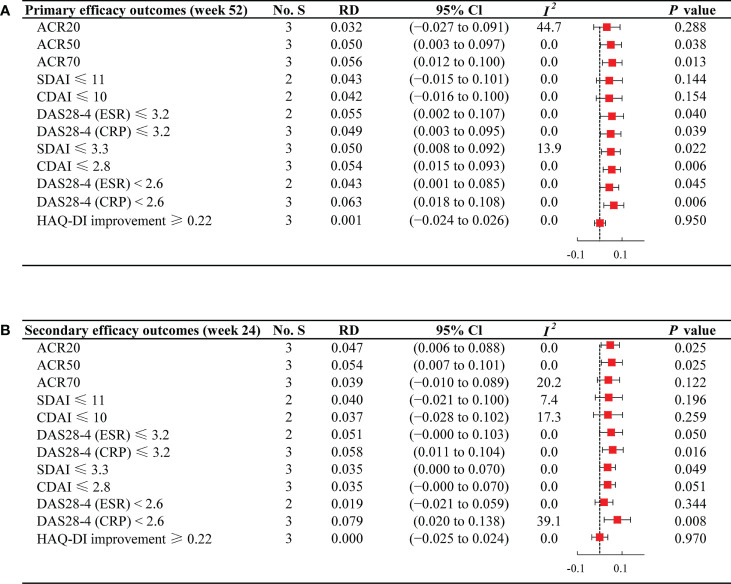
Forest plots of efficacy outcomes for JAKi combination therapy versus JAKi monotherapy **(A)** Forest plots of efficacy outcomes at week 52; **(B)** Forest plots of efficacy outcomes at week 24; No. S, numbers of studies; RD, risk difference; CI, confidence interval; *I^2^*, heterogeneity; ACR, American College of Rheumatology criteria; SDAI, Simplified Disease Activity Index; CDAI, Clinical Disease Activity Index; DAS28-4(ESR), Disease Activity Score in 28 joints, erythrocyte sedimentation rate; DAS28-4(CRP), Disease Activity Score in 28 joints, C-reactive protein; HAQ-DI, Health Assessment Questionnaire disability index.

The proportions of patients who achieved ACR response, HAQ-DI improvement ≥ 0.22, low disease activity, and remission were used to assess the efficacy of JAKi plus MTX versus JAKi alone. Forest plots of efficacy outcomes are shown in **Figure 2A**. In general, JAKi combination therapy was superior to JAKi monotherapy. The heterogeneity of the included studies was relatively low (*I^2^* < 50% for each outcome).

#### ACR and HAQ-DI

Viewed separately, at week 52, JAKi plus MTX was associated with a comparable ACR20 response rate to JAKi alone (RD 0.032; 95% CI −0.027 to 0.091). The proportion of patients achieving ACR20 was 72.69% (732/1007) in the combination group and 67.74% (510/753) in the JAKi group (**Supplementary Table 3**). For ACR50 and ACR70, patients receiving JAKi and MTX (ACR50: 56.72%; ACR70: 40.40%) attained a higher response rate compared with those receiving JAKi alone (ACR50: 49.26%; ACR70: 32.26%), with RD 0.050 (95% CI: 0.003 to 0.097) and 0.056 (95% CI: 0.012 to 0.100), respectively. Notably, no significant difference between the two regimens was found for the proportion of patients achieving HAQ-DI improvement ≥ 0.22 (RD 0.001; 95% CI −0.024 to 0.026).

#### Low disease activity and remission achievement

Regarding low disease activity at week 52, JAKi plus MTX regimen group presented a relatively higher percentage of patients achieving SDAI ≤ 11 (54.15%) and CDAI ≤ 10 (54.31%) than the JAKi monotherapy group (SDAI ≤ 11: 48.80%; CDAI ≤ 10: 49.17%), indicating more significant improvement in SDAI ≤ 11 (RD 0.043; 95% CI −0.015 to 0.101) and CDAI ≤ 10 (RD 0.042; 95% CI −0.016 to 0.100). Furthermore, significant differences were observed in the proportions of patients attaining DAS28-4 (ESR) ≤ 3.2 (RD 0.055; 95% CI 0.002 to 0.107) and DAS28-4 (CRP) ≤ 3.2 (RD 0.049; 95% CI 0.003 to 0.095) between the two groups. Regarding the percentage of patients attaining remission at week 52, we observed higher response rates among those treated with combination therapy compared with JAKi monotherapy, with RD 0.050 (95% CI 0.008 to 0.092) for SDAI ≤ 3.3, RD 0.054 (95% CI 0.015 to 0.093) for CDAI ≤ 2.8, RD 0.043 (95% CI 0.001 to 0.085) for DAS28-4 (ESR) < 2.6, and RD 0.063 (95% CI 0.018 to 0.108) for DAS28-4 (CRP) < 2.6.

### Secondary efficacy outcomes

The secondary endpoints at week 24 remained consistent with the primary outcomes, as presented in **Figure 2B**. Compared with JAKi monotherapy, patients treated with combination therapy at week 24 yielded higher percentages of response rates for ACR20 (RD 0.047; 95% CI 0.006 to 0.088) and ACR50 (RD 0.054; 95% CI 0.007 to 0.101). For ACR70, JAKi with MTX resulted in high response rates, although there was no significant difference between the two groups (RD 0.039; 95% CI −0.010 to 0.089). For the proportion of patients achieving HAQ-DI improvement ≥ 0.22 at week 24, patients under JAKi plus MTX or JAKi alone showed a similar trend (RD 0.000; 95% CI −0.025 to 0.024). The proportions of patients achieving low disease activity and remission was largely unchanged from week 24 to week 52.

### Safety outcomes

**Figure 3:**
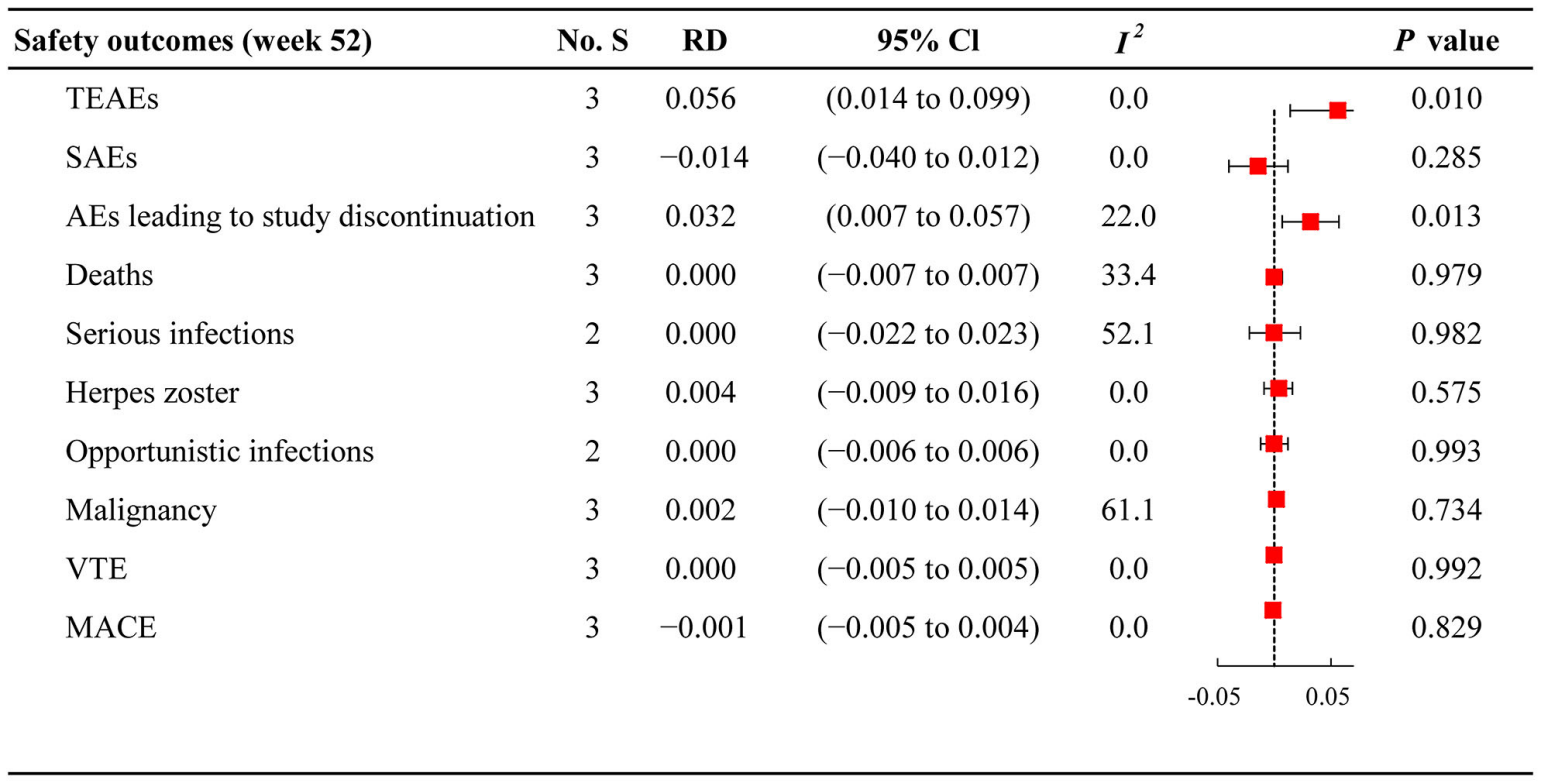
Forest plots of safety outcomes for JAKi combination therapy versus JAKi monotherapy No. S, numbers of studies; RD, risk difference; CI, confidence interval; *I^2^*, heterogeneity; TEAEs, treatment-emergent adverse events; SAEs, serious adverse events; AEs, adverse events; VTE, venous thromboembolism; MACE, major adverse cardiovascular events.

The meta-analysis assessed the tolerability of JAKi monotherapy and combination therapy in patients with active RA (**Figure 3** **)**. The incidence of TEAEs was 59.19% (596/1007) in the JAKi plus MTX group and 47.41% (357/753) in the JAKi group (**Supplementary Table 4**), indicating a higher risk of TEAEs within combination therapy versus JAKi monotherapy (RD 0.056; 95% CI 0.014 to 0.099). In addition, more patients reported AEs leading to study discontinuation in JAKi plus MTX group (7.65%) than in JAKi alone group (4.91%), with RD 0.032 (95% CI 0.007 to 0.057). However, the incidence of SAEs and deaths were similar between the two treatment groups (SAEs: RD −0.014; 95% CI −0.040 to 0.012 and deaths: RD 0.000; 95% CI −0.007 to 0.007). For adverse events of special interest, low frequencies were observed for both treatment groups in terms of serious infections, herpes zoster infection, opportunistic infections, malignancy, VTE, and MACE. The risk for serious infections was not higher in the combination group than in the JAKi group (RD 0.000; 95% CI –0.022 to 0.023). Both JAKi combination and JAKi monotherapy groups displayed similar risks of herpes zoster infection (RD 0.004; 95% CI –0.009 to 0.016), opportunistic infections (RD 0.000; 95% CI –0.006 to 0.006), malignancy (RD 0.002; 95% CI –0.010 to 0.014), VTE (RD 0.000; 95% CI –0.005 to 0.005), and MACE (RD –0.001; 95% CI –0.005 to 0.004).

## Discussion

To the best of our knowledge, the present study is the first to directly evaluate the effectiveness and safety of Janus kinase inhibitors treatment with or without MTX in patients with active RA. The JAKi plus MTX regimen showed superiority in achieving ACR response, HAQ-DI improvement, low disease activity, and remission versus JAKi alone. Furthermore, the JAKi combination therapy was associated with similar tolerability compared to monotherapy, except for TEAEs and AEs leading to study discontinuation.

According to the present recommendations for RA treatment, JAKi should be adopted as an add-on strategy to the ongoing csDMARD regimen, mostly MTX (2, 3). However, new evidence has demonstrated that JAKi with or without MTX presented favorable efficacy and acceptable safety compared with placebo or MTX. A meta-analysis identified that compared with placebo, JAKi monotherapy or combination therapy improved RA control as determined by ACR20 (risk ratio: 2.03) and HAQ-DI scores (mean differences: −0.31) (31). Concerning JAKi monotherapy, different JAK inhibitors led to statistically significant improvement in ACR20 response rate compared with placebo (odds ratio: from 2.03 to 17.24) (32, 33). Furthermore, the ACR50 and ACR70 response rates showed a similar trend to the ACR20 criteria. Compared with MTX, JAKi monotherapy also displayed superiority in RA improvement (20, 21). In terms of combination therapy, several RCTs had suggested that JAKi combined with MTX was preferred over MTX alone for different efficacy endpoints, including ACR response criteria and improvement in physical functioning (4, 5, 9, 21, 34, 35). Moreover, long-term extension studies determined that JAKi, in general, showed a consistent safety profile as monotherapy or combination therapy (36, 37).

However, in clinical practice, adherence to MTX is relatively low. A cross-sectional survey showed that forgetting to take MTX, thinking it was not needed when they felt well, and the concern about the long-term safety of MTX, contributed to the nonadherence to MTX (38). Gastrointestinal events are the most commonly reported AEs (pooled prevalence: 32.7%) in patients with RA starting MTX (39). Furthermore, the reluctance of patients to take multiple drugs to manage their disease is due to fear of drug-drug interactions or inadequate pharmacological clearance in elderly patients (40). Therefore, there is an urgent need for evidence to clarify whether JAKi monotherapy regimens could replace combination regimens to avoid inadequately controlled disease in some RA patients under JAKi monotherapy.

However, there have been few comparisons between JAKi monotherapy and combination therapy. A network meta-analysis demonstrated that several novel DMARDs (including tocilizumab, anti-tumor necrosis factor, and tofacitinib) showed different levels of efficacy as monotherapy and in combination with MTX (16). For example, tocilizumab combined with MTX displayed similar ACR responses versus tocilizumab alone, whereas anti-tumor necrosis factor or tofacitinib showed greater ACR response rates in combination regimen versus monotherapy. However, this study involved only a single JAKi (tofacitinib) and less effective evaluation indexes. Furthermore, it did not compare the safety profiles of JAKi monotherapy and combination therapy. In 2018, a systematic review assessing the efficacy of biologics and JAKi reported the superiority of bDMARDs/tsDMARDs + csDMARDs to bDMARDs/tsDMARDs monotherapy, without any individualized or fully quantified results for JAKi (17). Similarly, no safety outcomes were reported for the different treatment regimens.

Given the above limitations and uncertainty, it is necessary to rigorously assess the efficacy and safety of JAKi with or without MTX. As a result, we performed a systematic review and meta-analysis based on evidence from RCTs to comprehensively evaluate this issue using multiple indexes. Better treatment outcomes are usually achieved with combination therapy, and this was validated by our analysis. We determined that JAKi combination therapy with MTX is likely to be more effective than JAKi treatment alone. Of importance, for each outcome analyzed, efficacy was basically consistent at week 24 and week 52 regardless of whether JAKi was administered with or without MTX.

Clinically meaningful improvements in RA manifestations as measured by ACR response rates were maintained over time in patients who received JAKi as monotherapy or combination therapy. The results of the present analysis were in line with those of a previous conducted network meta-analysis, in which greater ACR20/50/70 responses were observed with tofacitinib plus MTX than with tofacitinib monotherapy at week 24 (the probability that tofacitinib + MTX was better than tofacitinib at attaining ACR 20/50/70 responses was 83%, 84%, and 94%, respectively) (16). Although ACR response is a composite measure that captures improvement in tender and swollen joint, achieving remission or low disease activity, as well as improvement in physical functioning measured by HAQ-DI, are the main goals of RA treatment (2, 3). Our analysis demonstrated that in patients with active RA, JAKi, in combination with MTX, was more prone to attain remission and low disease activity than JAKi monotherapy, although most indexes were associated with a favorable trend of response rate without a significant difference. Regarding the HAQ-DI improvement, interestingly, JAKi monotherapy displayed comparable efficacy to combination therapy at week 24 and week 52. A pooled analysis of tofacitinib showed consistent results and was maintained until month 72, albeit with a loss of direct comparison between monotherapy and combination therapy (41).

As a new targeted drug class, JAKi may affect broader pathways beyond those being targeted, so evidence of the safety profile of JAKi still needs careful investigation and accumulation in patients with RA (12, 15). The incidence of adverse events seems similar to bDMARDs, with infections (42), VTE (43, 44), malignancies (45), and cardiovascular safety (46, 47) being the most concerning adverse events (48). In terms of the safety of JAKi with or without MTX, the frequency of adverse events was comparable between the two regimens, except for TEAEs and AEs leading to study discontinuation. This seems reasonable because of the intolerance and nonadherence to MTX in some patients; therefore, JAKi monotherapy appears to be a preferred treatment option for such patients (17, 38). The results confirm the credibility of our analysis. Regarding adverse events of particular interest (serious infections, malignancy, VTE, MACE, etc.), no meaningful difference was observed between JAKi monotherapy and combination therapy; however, these results should be interpreted with caution because of the limited number of adverse events assessed.

In addition, in our meta-analysis, for the remaining double-arm-zero-events in the included trials, we used risk difference and the Mantel-Haenszel random-effects model to analyze all events in patients because double-arm-zero-events studies are automatically discarded when the Mantel-Haenszel method with odds ratio or risk ratio is utilized (49). Omitting double-arm-zero-events would contribute to inappropriate choices in evidence synthesis, and misleading the healthcare practice; therefore, the Mantel-Haenszel random-effects model along with risk difference would be a favorable choice.

### Strengths and limitations

Our study offers the first comprehensive and detailed comparative evaluation of the effectiveness of JAKi monotherapy versus combination therapy with MTX based on various effective outcomes (ACR response rate, HAQ-DI improvement, low disease activity, and remission achievement) and follow-up analyses (week 24 and week 52), as well as the safety of the two therapeutic regimens. Although the number of studies included for comparison was too small to generate an absolute conclusion, in contrast to individual studies, our analysis obtained more accurate data by increasing the statistical power and validated the superiority of JAKi combination therapy to monotherapy with comparable tolerability in patients with RA. However, the analysis reported here has several limitations. First, the study covered a minimal number of studies involving only three JAKi agents (tofacitinib, baricitinib, and filgotinib). Other JAKi, such as upadacitinib and peficitinib were not included due to current drug trials (upadacitinib, peficitinib) failing to address the comparison as monotherapy and in combination with MTX. The limited studies made subgroup analysis unavailable, except for effective outcomes by different follow-ups (week 24 and week 52). Specifically, the low frequency of VTE or malignancy was insufficient to determine the safety outcomes of JAKi as monotherapy or combination therapy. Accumulation of evidence of more RCTs about other JAKi in decision-making is needed. Second, the limited number of articles included prevented a full stratification for disease sub-populations of interest, like csDMARDs naïve or csDMARDs-experienced patients. Third, an analysis of approximately one-year RCT data would provide a somewhat limited picture as it is insufficient to determine the long-term durability and tolerability of JAKi monotherapy and combination therapy with MTX. Meanwhile, radiographic progression of RA patients was not assessed. More comparative studies and long-term observational studies are warranted in the future. Furthermore, the results of this meta-analysis were based on evidence from RCTs and may not be generalizable to the broader RA population with heterogeneous characteristics and therapeutic regimens in the real world.

## Conclusion

Based on a meta-analysis of direct comparisons of RCT data, we found that JAKi, in combination with MTX, demonstrated superiority in ACR responses, low disease activity, and remission achievement, to JAKi monotherapy in active RA treatment. The two regimens presented comparable physical functioning measured by HAQ-DI improvement, along with similar tolerability, except for high risks of TEAEs and AEs leading to study discontinuation in combination therapy. Long-term pharmacovigilance of JAKi as monotherapy and in combination with MTX in RA is warranted.

## Data availability statement

The original contributions presented in the study are included in the article/**Supplementary Material**. Further inquiries can be directed to the corresponding author.

## Author contributions

All authors made substantial contributions to the conception and design of this study. LL and F-HS drafted the manuscript. LL and Y-DY performed the meta-analysis. JL, Z-CG and H-WL revised and approved the final manuscript. All authors contributed to the article and approved the submitted version.

## Funding

This study was supported by the Clinical Research Innovation and Cultivation Fund of Ren Ji hospital (RJPY-LX-008), Ren Ji Boost Project of National Natural Science Foundation of China (RJTJ-JX-001), Research Project of Drug Clinical Comprehensive Evaluation and Drug Treatment Pathway (SHYXH-ZP-2021-001), and Clinical Research Training Project of Renji Hospital (PY2018-III-04).

## Conflict of interest

The authors declare that the research was conducted in the absence of any commercial or financial relationships that could be construed as a potential conflict of interest.

## Publisher’s note

All claims expressed in this article are solely those of the authors and do not necessarily represent those of their affiliated organizations, or those of the publisher, the editors and the reviewers. Any product that may be evaluated in this article, or claim that may be made by its manufacturer, is not guaranteed or endorsed by the publisher.
