# Supplementary material for: Comparative efficacy and safety of JAK inhibitors as monotherapy and in combination with methotrexate in patients with active rheumatoid arthritis: A systematic review and meta-analysis
Source: Front Immunol. 2022 Sep 29;13:977265. doi: 10.3389/fimmu.2022.977265 (PMC9556706; doi:10.3389/fimmu.2022.977265)
Supplement: Supplementary file 1 [file DataSheet_1.docx]

**Title:** Comparative efficacy and safety of JAK inhibitors as monotherapy and in combination with methotrexate in patients with active rheumatoid arthritis: A meta-analysis of randomized controlled trials

[Supplementary Tables 3](#_Toc113795451)

[Table S1 Search strategy used in May 17, 2022 3](#_Toc113795452)

[Table S2 Excluded studies with reasons 5](#_Toc113795453)

[Table S3 Summarized efficacy outcomes in the included studies 7](#_Toc113795454)

[Table S4 Summarized adverse events in the included studies 8](#_Toc113795455)

[Table S5 PRISMA checklists 9](#_Toc113795456)

[Supplementary Figures 12](#_Toc113795457)

[Figure S1 Forest plots of primary efficacy outcomes (week 52) for JAKi combination therapy versus JAKi monotherapy 12](#_Toc113795458)

[Figure S2 Forest plots of secondary efficacy outcomes (week 24) for JAKi combination therapy versus JAKi monotherapy 13](#_Toc113795459)

[Figure S3 Forest plots of safety outcomes for JAKi combination therapy versus JAKi monotherapy 14](#_Toc113795460)

[References of excluded studies 15](#_Toc113795461)

**Supplementary Tables**

**Table S1 Search strategy used in May 17, 2022**

| **Literature databases** | **Search items** | **Items found** |
| --- | --- | --- |
| Medline | (((rheumatoid arthritis[Title/Abstract]) OR (RA[Title/Abstract])) AND ((((((((((((((((((((JAKi[Title/Abstract]) OR (Janus kinase inhibitor[Title/Abstract])) OR (tofacitinib[Title/Abstract])) OR (Xeljanz[Title/Abstract])) OR (XELJANZ XR[Title/Abstract])) OR (CP-690550[Title/Abstract])) OR (LY3009104[Title/Abstract])) OR (tasocitinib[Title/Abstract])) OR (baricitinib[Title/Abstract])) OR (Olumiant[Title/Abstract])) OR (LY-3009104[Title/Abstract])) OR (INCB-028050[Title/Abstract])) OR (upadacitinib[Title/Abstract])) OR (Rinvoq[Title/Abstract])) OR (ABT-494[Title/Abstract])) OR (filgotinib[Title/Abstract])) OR (GLPG0634[Title/Abstract])) OR (GS-6034[Title/Abstract])) OR (peficitinib[Title/Abstract])) OR (ASP015K[Title/Abstract]))) AND (((randomized controlled trial) OR (controlled clinical trial)) OR (clinical trial)) | 298 |
| Embase | ('rheumatoid arthritis':ti,ab,kw OR ra:ti,ab,kw) AND (jaki:ti,ab,kw OR 'janus kinase inhibitor':ti,ab,kw OR tofacitinib:ti,ab,kw OR xeljanz:ti,ab,kw OR 'xeljanz xr':ti,ab,kw OR 'cp 690550':ti,ab,kw OR ly3009104:ti,ab,kw OR tasocitinib:ti,ab,kw OR baricitinib:ti,ab,kw OR olumiant:ti,ab,kw OR 'ly 3009104':ti,ab,kw OR 'incb 028050':ti,ab,kw OR upadacitinib:ti,ab,kw OR rinvoq:ti,ab,kw OR 'abt 494':ti,ab,kw OR filgotinib:ti,ab,kw OR glpg0634:ti,ab,kw OR 'gs 6034':ti,ab,kw OR peficitinib:ti,ab,kw OR asp015k:ti,ab,kw) AND (randomized AND controlled AND trial OR (controlled AND clinical AND trial) OR (clinical AND trial)) AND [embase]/lim NOT ([embase]/lim AND [medline]/lim) | 1,130 |
| Cochrane | ((rheumatoid arthritis):ti,ab,kw OR (RA):ti,ab,kw) AND (JAKi):ti,ab,kw OR(janus kinase inhibitor):ti,ab,kw OR (tofacitinib):ti,ab,kw OR (xeljanz):ti,ab,kw OR (xeljanz xr):ti,ab,kw OR (cp 690550):ti,ab,kw OR (ly3009104):ti,ab,kw OR (tasocitinib):ti,ab,kw OR (baricitinib):ti,ab,kw OR (Olumiant):ti,ab,kw OR (ly 3009104):ti,ab,kw OR (incb 028050):ti,ab,kw OR (upadacitinib):ti,ab,kw OR (rinvoq):ti,ab,kw OR (abt 494):ti,ab,kw OR (filgotinib):ti,ab,kw OR (glpg0634):ti,ab,kw OR (gs 6034):ti,ab,kw OR (peficitinib):ti,ab,kw OR (asp015k):ti,ab,kw) AND (randomized controlled trial OR (controlled clinical trial) OR (clinical trial)) | 912 |
| Overall |  | 2,340 |
| Duplicates |  | 793 |

**Table S2 Excluded studies with reasons**

| **Number** | **Drugs** | **Study** | **Reason for exclusion** |
| --- | --- | --- | --- |
| 1 | Tofacitinib | Burmester 2013 [1] | Not report JAKi monotherapy |
| 2 | Tofacitinib | Cohen 2019 [2] | Open-label study |
| 3 | Tofacitinib | Fleischmann 2012 [3] | Not report JAKi plus MTX |
| 4 | Tofacitinib | Fleischmann 2012 [4] | Not report JAKi plus MTX |
| 5 | Tofacitinib | Kremer 2012 [5] | Not report JAKi monotherapy |
| 6 | Tofacitinib | Kremer 2013 [6] | Not report JAKi monotherapy |
| 7 | Tofacitinib | Kremer 2015 [7] | Phase 1 study |
| 8 | Tofacitinib | Mcinnes 2014 [8] | Not report JAKi plus MTX |
| 9 | Tofacitinib | Nakamura 2018 [9] | Open-label study |
| 10 | Tofacitinib | Tanaka 2011 [10] | Not report JAKi monotherapy |
| 11 | Tofacitinib | Tanaka 2015 [11] | Not report JAKi plus MTX |
| 12 | Tofacitinib | Van der Heijde 2019 [12] | Not report JAKi monotherapy |
| 13 | Tofacitinib | Vollenhoven 2012 [13] | Not report JAKi monotherapy |
| 14 | Tofacitinib | Winthrop 2017 [14] | Not report JAKi monotherapy |
| 15 | Tofacitinib | Wollenhaupt 2019 [15] | Long-term extension study |
| 16 | Tofacitinib | Yamanaka 2016 [16] | Long-term extension study |
| 17 | Baricitinib | Dougados 2017 [17] | Not report JAKi monotherapy |
| 18 | Baricitinib | Genovese 2016 [18] | Not report JAKi plus MTX |
| 19 | Baricitinib | Keystone 2015 [19] | Not report JAKi monotherapy |
| 20 | Baricitinib | Keystone 2018 [20] | Long-term extension study |
| 21 | Baricitinib | Takeuchi 2019 [21] | Long-term extension study |
| 22 | Baricitinib | Tanaka 2016 [22] | Not report JAKi monotherapy |
| 23 | Baricitinib | Tanaka 2018 [23] | Long-term extension study |
| 24 | Baricitinib | Taylor 2017 [24] | Not report JAKi monotherapy |
| 25 | Filgotinib | Combe 2021 [25] | Not report JAKi monotherapy |
| 26 | Filgotinib | Genovese 2019 [26] | Not report JAKi monotherapy |
| 27 | Filgotinib | Kavanaugh 2017 [27] | Not report JAKi plus MTX |
| 28 | Filgotinib | Westhovens 2017 [28] | Not report JAKi monotherapy |
| 29 | Peficitinib | Genovese 2017 [29] | Not report JAKi plus MTX |
| 30 | Peficitinib | Genovese 2019 [30] | Long-term extension study |
| 31 | Peficitinib | Kivitz 2017 [31] | Not report JAKi monotherapy |
| 32 | Peficitinib | Takeuchi 2016 [32] | Not report JAKi plus MTX |
| 33 | Peficitinib | Takeuchi 2019 [33] | Not report JAKi monotherapy |
| 34 | Peficitinib | Takeuchi 2020 [34] | Long-term extension study |
| 35 | Peficitinib | Takeuchi 2021 [35] | Long-term extension study |
| 36 | Peficitinib | Tanaka 2019 [36] | Not report JAKi plus MTX |
| 37 | Upadacitinib | Burmester 2018 [37] | Not report JAKi monotherapy |
| 38 | Upadacitinib | Fleischmann 2019 [38] | Not report JAKi monotherapy |
| 39 | Upadacitinib | Fleischmann 2019 [39] | Not report JAKi monotherapy |
| 40 | Upadacitinib | Genovese 2016 [40] | Not report JAKi monotherapy |
| 41 | Upadacitinib | Genovese 2018 [41] | Not report JAKi monotherapy |
| 42 | Upadacitinib | Kameda 2020 [42] | Not report JAKi monotherapy |
| 43 | Upadacitinib | Kameda 2021 [43] | Not report JAKi plus MTX |
| 44 | Upadacitinib | Kremer 2016 [44] | Not report JAKi monotherapy |
| 45 | Upadacitinib | Rubbert-Roth 2020 [45] | Not report JAKi plus MTX |
| 46 | Upadacitinib | Smolen 2019 [46] | Not report JAKi plus MTX |
| 47 | Upadacitinib | Vollenhoven 2020 [47] | Not report JAKi plus MTX |

JAKi, Janus kinase inhibitors; MTX, methotrexate.

**Table S3 Summarized efficacy outcomes in the included studies**

| **Efficacy outcomes** | **JAKi + MTX (n=1,007)** | **JAKi (n=753)** |
| --- | --- | --- |
| ACR20 | 732 (72.69%) | 510 (67.74%) |
| ACR50 | 571 (56.72%) | 371 (49.26%) |
| ACR70 | 407 (40.40%) | 243 (32.26%) |
| SDAI ≤ 11* | 320 (54.15%) | 265 (48.80%) |
| CDAI ≤ 10* | 321 (54.31%) | 267 (49.17%) |
| DAS28-4 (ESR) ≤ 3.2* | 198 (33.50%) | 145 (26.70%) |
| DAS28-4 (CRP) ≤ 3.2 | 598 (59.39%) | 386 (51.26%) |
| SDAI ≤ 3.3 | 272 (27.01%) | 146 (19.39%) |
| CDAI ≤ 2.8 | 276 (27.43%) | 149 (19.79%) |
| DAS28-4 (ESR) < 2.6* | 118 (19.97%) | 78 (14.36%) |
| DAS28-4 (CRP) < 2.6 | 441 (43.81%) | 259 (34.40%) |
| HAQ-DI improvement ≥ 0.22 | 798 (79.25%) | 553 (73.44%) |

Data are *n* (%), with *n* presented the number of patients in that group. * FINCH 3 did not report the related outcomes, so the total number of patients in the remaining trials was 591 in JAKi + MTX group and 543 in JAKi group, respectively.

**Table S4 Summarized adverse events in the included studies**

| **Adverse events** | **JAKi + MTX (n=1,007)** | **JAKi (n=753)** |
| --- | --- | --- |
| TEAEs | 596 (59.19%) | 357 (47.41%) |
| SAEs | 70 (6.95%) | 64 (8.50%) |
| AEs leading to study discontinuation | 77 (7.65%) | 37 (4.91%) |
| Deaths | 3 (0.30%) | 2 (0.27%) |
| Serious infections* | 15 (1.89%) | 11 (1.85%) |
| Herpes zoster | 19 (1.89%) | 12 (1.59%) |
| Opportunistic infections* | 2 (0.25%) | 2 (0.34%) |
| Malignancy | 7 (0.70%) | 4 (0.53%) |
| VTE | 1 (0.10%) | 1 (0.13%) |
| MACE | 4 (0.40%) | 3 (0.40%) |

Data are *n* (%), with *n* presented the number of patients occurred the adverse events in that group.

TEAEs, treatment-emergent adverse events; SAEs, serious adverse events; AEs, adverse events; VTE, venous thromboembolism; MACE, major adverse cardiovascular events.

* RA-BEGIN did not report the related adverse events, so the total number of patients in the remaining trials was 792 in JAKi + MTX group and 594 in JAKi group, respectively.

**Table S5 PRISMA checklists**

| **Section and Topic** | **Item #** | **Checklist item** | **Location where item is reported** |
| --- | --- | --- | --- |
| **TITLE** | | |  |
| Title | 1 | Identify the report as a systematic review. | P1 |
| **ABSTRACT** | | |  |
| Abstract | 2 | See the PRISMA 2020 for Abstracts checklist. | P2 |
| **INTRODUCTION** | | |  |
| Rationale | 3 | Describe the rationale for the review in the context of existing knowledge. | P3 |
| Objectives | 4 | Provide an explicit statement of the objective(s) or question(s) the review addresses. | P3-4 |
| **METHODS** | | |  |
| Eligibility criteria | 5 | Specify the inclusion and exclusion criteria for the review and how studies were grouped for the syntheses. | P5 |
| Information sources | 6 | Specify all databases, registers, websites, organisations, reference lists and other sources searched or consulted to identify studies. Specify the date when each source was last searched or consulted. | P5 |
| Search strategy | 7 | Present the full search strategies for all databases, registers and websites, including any filters and limits used. | P5 |
| Selection process | 8 | Specify the methods used to decide whether a study met the inclusion criteria of the review, including how many reviewers screened each record and each report retrieved, whether they worked independently, and if applicable, details of automation tools used in the process. | P5 |
| Data collection process | 9 | Specify the methods used to collect data from reports, including how many reviewers collected data from each report, whether they worked independently, any processes for obtaining or confirming data from study investigators, and if applicable, details of automation tools used in the process. | P5-6 |
| Data items | 10a | List and define all outcomes for which data were sought. Specify whether all results that were compatible with each outcome domain in each study were sought (e.g. for all measures, time points, analyses), and if not, the methods used to decide which results to collect. | P5-6 |
|  | 10b | List and define all other variables for which data were sought (e.g. participant and intervention characteristics, funding sources). Describe any assumptions made about any missing or unclear information. | P5-6 |
| Study risk of bias assessment | 11 | Specify the methods used to assess risk of bias in the included studies, including details of the tool(s) used, how many reviewers assessed each study and whether they worked independently, and if applicable, details of automation tools used in the process. | P5-6 |
| Effect measures | 12 | Specify for each outcome the effect measure(s) (e.g. risk ratio, mean difference) used in the synthesis or presentation of results. | P5-6 |
| Synthesis methods | 13a | Describe the processes used to decide which studies were eligible for each synthesis (e.g. tabulating the study intervention characteristics and comparing against the planned groups for each synthesis (item #5)). | P6 |
|  | 13b | Describe any methods required to prepare the data for presentation or synthesis, such as handling of missing summary statistics, or data conversions. | P6 |
|  | 13c | Describe any methods used to tabulate or visually display results of individual studies and syntheses. | P6 |
|  | 13d | Describe any methods used to synthesize results and provide a rationale for the choice(s). If meta-analysis was performed, describe the model(s), method(s) to identify the presence and extent of statistical heterogeneity, and software package(s) used. | P6 |
|  | 13e | Describe any methods used to explore possible causes of heterogeneity among study results (e.g. subgroup analysis, meta-regression). |  |
|  | 13f | Describe any sensitivity analyses conducted to assess robustness of the synthesized results. | P6 |
| Reporting bias assessment | 14 | Describe any methods used to assess risk of bias due to missing results in a synthesis (arising from reporting biases). | P6 |
| Certainty assessment | 15 | Describe any methods used to assess certainty (or confidence) in the body of evidence for an outcome. | P5-6 |
| **RESULTS** | | |  |
| Study selection | 16a | Describe the results of the search and selection process, from the number of records identified in the search to the number of studies included in the review, ideally using a flow diagram. | P6 |
|  | 16b | Cite studies that might appear to meet the inclusion criteria, but which were excluded, and explain why they were excluded. | P6 |
| Study characteristics | 17 | Cite each included study and present its characteristics. | P6-7 |
| Risk of bias in studies | 18 | Present assessments of risk of bias for each included study. | P7 |
| Results of individual studies | 19 | For all outcomes, present, for each study: (a) summary statistics for each group (where appropriate) and (b) an effect estimate and its precision (e.g. confidence/credible interval), ideally using structured tables or plots. | P7-9 |
| Results of syntheses | 20a | For each synthesis, briefly summarise the characteristics and risk of bias among contributing studies. | P7-9 |
|  | 20b | Present results of all statistical syntheses conducted. If meta-analysis was done, present for each the summary estimate and its precision (e.g. confidence/credible interval) and measures of statistical heterogeneity. If comparing groups, describe the direction of the effect. | P7-9 |
|  | 20c | Present results of all investigations of possible causes of heterogeneity among study results. | P7-9 |
|  | 20d | Present results of all sensitivity analyses conducted to assess the robustness of the synthesized results. | P7-9 |
| Reporting biases | 21 | Present assessments of risk of bias due to missing results (arising from reporting biases) for each synthesis assessed. | P7-9 |
| Certainty of evidence | 22 | Present assessments of certainty (or confidence) in the body of evidence for each outcome assessed. | P7-9 |
| **DISCUSSION** | | |  |
| Discussion | 23a | Provide a general interpretation of the results in the context of other evidence. | P9 |
|  | 23b | Discuss any limitations of the evidence included in the review. | P9-12 |
|  | 23c | Discuss any limitations of the review processes used. | P9-12 |
|  | 23d | Discuss implications of the results for practice, policy, and future research. | P9-12 |
| **OTHER INFORMATION** | | |  |
| Registration and protocol | 24a | Provide registration information for the review, including register name and registration number, or state that the review was not registered. | P4 |
|  | 24b | Indicate where the review protocol can be accessed, or state that a protocol was not prepared. |  |
|  | 24c | Describe and explain any amendments to information provided at registration or in the protocol. |  |
| Support | 25 | Describe sources of financial or non-financial support for the review, and the role of the funders or sponsors in the review. | P13 |
| Competing interests | 26 | Declare any competing interests of review authors. | P13 |
| Availability of data, code and other materials | 27 | Report which of the following are publicly available and where they can be found: template data collection forms; data extracted from included studies; data used for all analyses; analytic code; any other materials used in the review. | P13 |

*From:*  Page MJ, McKenzie JE, Bossuyt PM, Boutron I, Hoffmann TC, Mulrow CD, et al. The PRISMA 2020 statement: an updated guideline for reporting systematic reviews. BMJ 2021;372:n71. doi: 10.1136/bmj.n71

For more information, visit: <http://www.prisma-statement.org/>

**Supplementary Figures**

**Figure S1 Forest plots of primary efficacy outcomes (week 52) for JAKi combination therapy versus JAKi monotherapy**


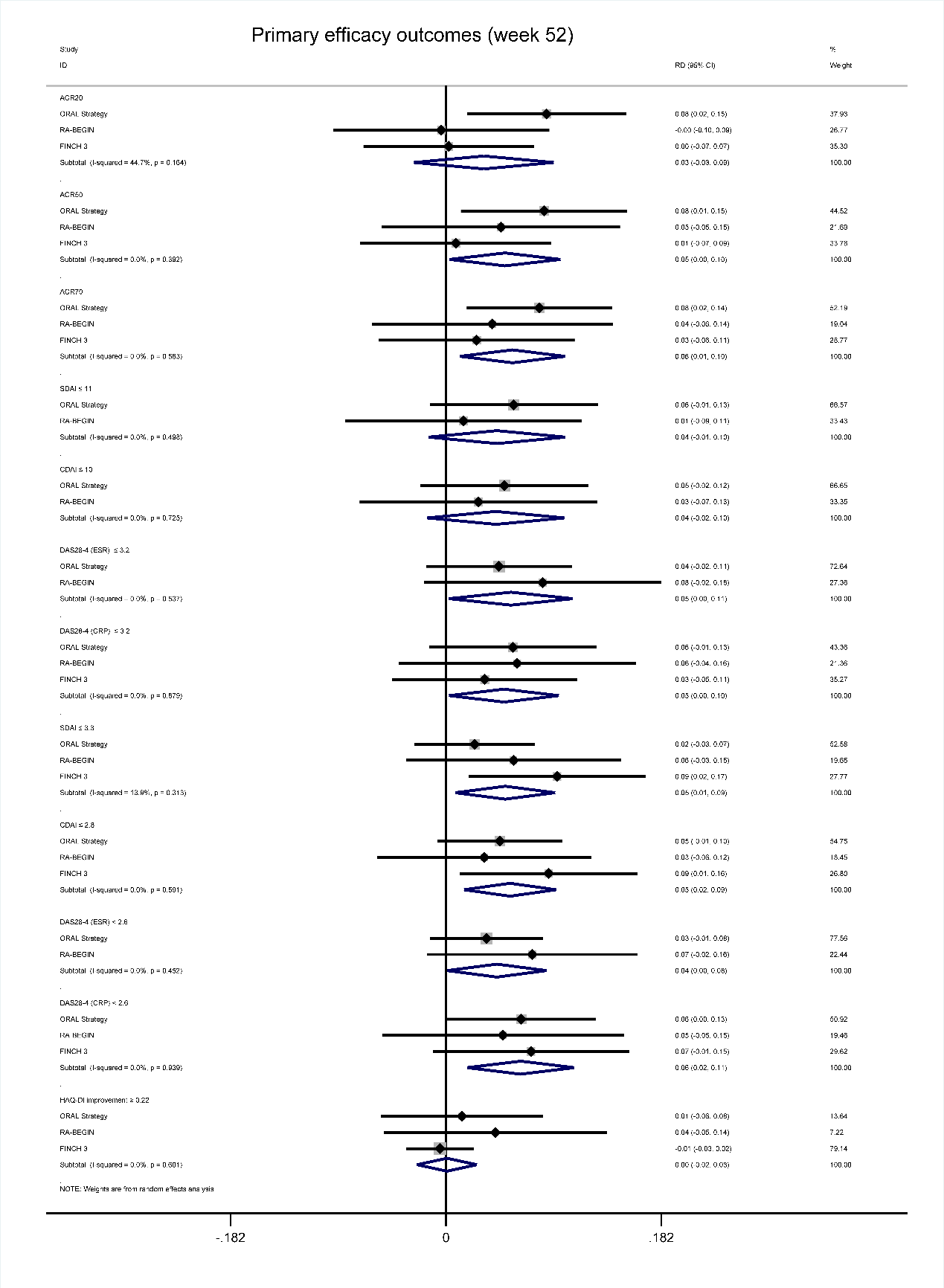


**Figure S2 Forest plots of secondary efficacy outcomes (week 24) for JAKi combination therapy versus JAKi monotherapy**


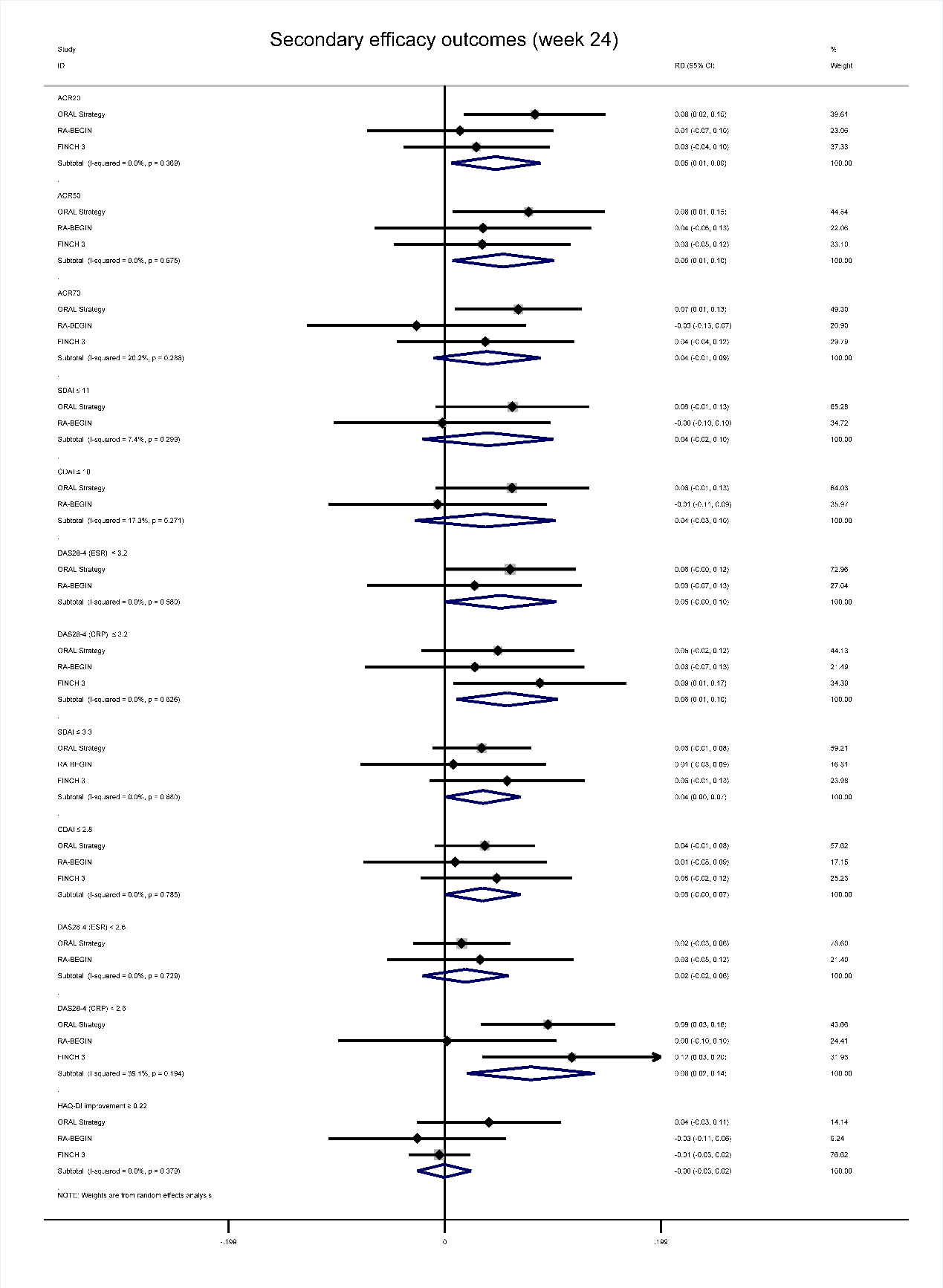


**Figure S3 Forest plots of safety outcomes for JAKi combination therapy versus JAKi monotherapy**


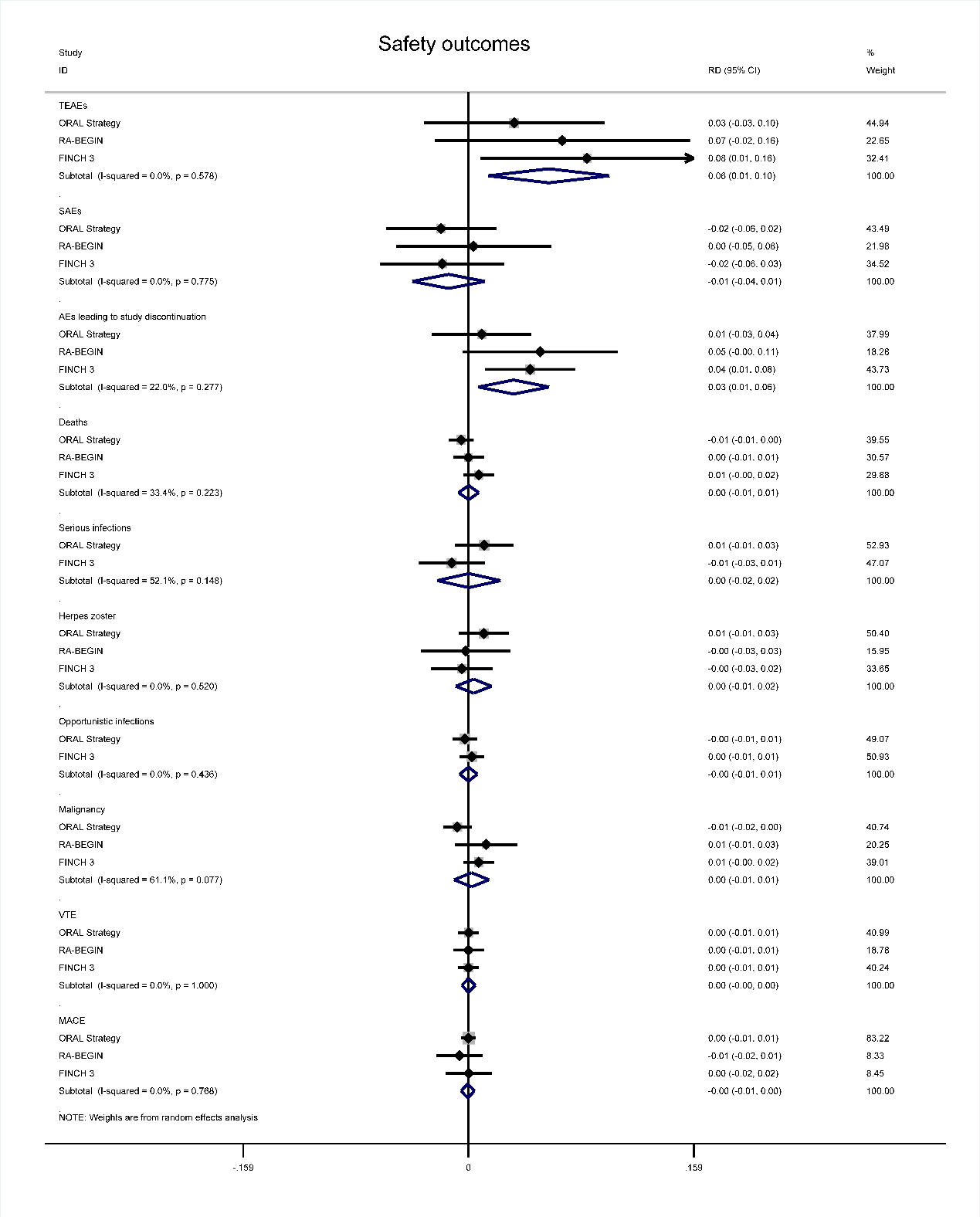


**References of excluded studies**

1. Burmester GR, Blanco R, Charles-Schoeman C, Wollenhaupt J, Zerbini C, Benda B, Gruben D, Wallenstein G, Krishnaswami S, Zwillich SH et al: Tofacitinib (CP-690,550) in combination with methotrexate in patients with active rheumatoid arthritis with an inadequate response to tumour necrosis factor inhibitors: a randomised phase 3 trial. Lancet (London, England) 2013, 381(9865):451‐460.

2. Cohen SB, Pope J, Haraoui B, Irazoque-Palazuelos F, Korkosz M, Diehl A, Rivas JL, Lukic T, Liu S, Stockert L et al: Methotrexate withdrawal in patients with rheumatoid arthritis who achieve low disease activity with tofacitinib modified-release 11 mg once daily plus methotrexate (ORAL Shift): a randomised, phase 3b/4, non-inferiority trial. The lancet rheumatology 2019, 1(1):e23‐e34.

3. Fleischmann R, Cutolo M, Genovese MC, Lee EB, Kanik KS, Sadis S, Connell CA, Gruben D, Krishnaswami S, Wallenstein G et al: Phase IIb dose-ranging study of the oral JAK inhibitor tofacitinib (CP-690,550) or adalimumab monotherapy versus placebo in patients with active rheumatoid arthritis with an inadequate response to disease-modifying antirheumatic drugs. Arthritis and rheumatism 2012, 64(3):617‐629.

4. Fleischmann R, Kremer J, Cush J, Schulze-Koops H, Connell CA, Bradley JD, Gruben D, Wallenstein GV, Zwillich SH, Kanik KS: Placebo-controlled trial of tofacitinib monotherapy in rheumatoid arthritis. New England journal of medicine 2012, 367(6):495‐507.

5. Kremer JM, Cohen S, Wilkinson BE, Connell CA, French JL, Gomez-Reino J, Gruben D, Kanik KS, Krishnaswami S, Pascual-Ramos V et al: A phase IIb dose-ranging study of the oral JAK inhibitor tofacitinib (CP-690,550) versus placebo in combination with background methotrexate in patients with active rheumatoid arthritis and an inadequate response to methotrexate alone. Arthritis and rheumatism 2012, 64(4):970-981.

6. Kremer J, Li ZG, Hall S, Fleischmann R, Genovese M, Martin-Mola E, Isaacs JD, Gruben D, Wallenstein G, Krishnaswami S et al: Tofacitinib in combination with nonbiologic disease-modifying antirheumatic drugs in patients with active rheumatoid arthritis: a randomized trial. Annals of internal medicine 2013, 159(4):253-261.

7. Kremer JM, Kivitz AJ, Simon-Campos JA, Nasonov EL, Tony HP, Lee SK, Vlahos B, Hammond C, Bukowski J, Li H et al: Evaluation of the effect of tofacitinib on measured glomerular filtration rate in patients with active rheumatoid arthritis: results from a randomised controlled trial. Arthritis research & therapy 2015, 17(1):95.

8. McInnes IB, Kim HY, Lee SH, Mandel D, Song YW, Connell CA, Luo Z, Brosnan MJ, Zuckerman A, Zwillich SH et al: Open-label tofacitinib and double-blind atorvastatin in rheumatoid arthritis patients: a randomised study. Annals of the rheumatic diseases 2014, 73(1):124-131.

9. Nakamura Y, Suzuki T, Yamazaki H, Kato H: Tofacitinib versus non-tumor necrosis factor biologics for patients with active rheumatoid arthritis. Archives of rheumatology 2018, 33(2):154‐159.

10. Tanaka Y, Suzuki M, Nakamura H, Toyoizumi S, Zwillich SH: Phase II study of tofacitinib (CP-690,550) combined with methotrexate in patients with rheumatoid arthritis and an inadequate response to methotrexate. Arthritis care & research 2011, 63(8):1150‐1158.

11. Tanaka Y, Takeuchi T, Yamanaka H, Nakamura H, Toyoizumi S, Zwillich S: Efficacy and safety of tofacitinib as monotherapy in Japanese patients with active rheumatoid arthritis: a 12-week, randomized, phase 2 study. Modern rheumatology 2015, 25(4):514‐521.

12. van der Heijde D, Strand V, Tanaka Y, Keystone E, Kremer J, Zerbini CAF, Cardiel MH, Cohen S, Nash P, Song YW et al: Tofacitinib in Combination With Methotrexate in Patients With Rheumatoid Arthritis: Clinical Efficacy, Radiographic, and Safety Outcomes From a Twenty-Four-Month, Phase III Study. Arthritis & rheumatology (Hoboken, NJ) 2019, 71(6):878-891.

13. van Vollenhoven RF, Fleischmann R, Cohen S, Lee EB, García Meijide JA, Wagner S, Forejtova S, Zwillich SH, Gruben D, Koncz T et al: Tofacitinib or adalimumab versus placebo in rheumatoid arthritis. The New England journal of medicine 2012, 367(6):508-519.

14. Winthrop KL, Wouters AG, Choy EH, Soma K, Hodge JA, Nduaka CI, Biswas P, Needle E, Passador S, Mojcik CF et al: The Safety and Immunogenicity of Live Zoster Vaccination in Patients With Rheumatoid Arthritis Before Starting Tofacitinib: A Randomized Phase II Trial. Arthritis & rheumatology (Hoboken, NJ) 2017, 69(10):1969-1977.

15. Wollenhaupt J, Lee EB, Curtis JR, Silverfield J, Terry K, Soma K, Mojcik C, Demasi R, Strengholt S, Kwok K et al: Safety and efficacy of tofacitinib for up to 9.5 years in the treatment of rheumatoid arthritis: final results of a global, open-label, long-term extension study. Arthritis research & therapy 2019, 21(1).

16. Yamanaka H, Tanaka Y, Takeuchi T, Sugiyama N, Yuasa H, Toyoizumi S, Morishima Y, Hirose T, Zwillich S: Tofacitinib, an oral Janus kinase inhibitor, as monotherapy or with background methotrexate, in Japanese patients with rheumatoid arthritis: an open-label, long-term extension study. Arthritis research & therapy 2016, 18:34.

17. Dougados M, van der Heijde D, Chen YC, Greenwald M, Drescher E, Liu J, Beattie S, Witt S, de la Torre I, Gaich C et al: Baricitinib in patients with inadequate response or intolerance to conventional synthetic DMARDs: results from the RA-BUILD study. Annals of the rheumatic diseases 2017, 76(1):88‐95.

18. Genovese MC, Kremer J, Zamani O, Ludivico C, Krogulec M, Xie L, Beattie SD, Koch AE, Cardillo TE, Rooney TP et al: Baricitinib in Patients with Refractory Rheumatoid Arthritis. New England journal of medicine 2016, 374(13):1243‐1252.

19. Keystone EC, Taylor PC, Drescher E, Schlichting DE, Beattie SD, Berclaz PY, Lee CH, Fidelus-Gort RK, Luchi ME, Rooney TP et al: Safety and efficacy of baricitinib at 24 weeks in patients with rheumatoid arthritis who have had an inadequate response to methotrexate. Annals of the rheumatic diseases 2015, 74(2):333-340.

20. Keystone EC, Genovese MC, Schlichting DE, de la Torre I, Beattie SD, Rooney TP, Taylor PC: Safety and Efficacy of Baricitinib Through 128 Weeks in an Open-label, Longterm Extension Study in Patients with Rheumatoid Arthritis. Journal of rheumatology 2018, 45(1):14‐21.

21. Takeuchi T, Genovese MC, Haraoui B, Li Z, Xie L, Klar R, Pinto-Correia A, Otawa S, Lopez-Romero P, de la Torre I et al: Dose reduction of baricitinib in patients with rheumatoid arthritis achieving sustained disease control: results of a prospective study. Annals of the rheumatic diseases 2019, 78(2):171-178.

22. Tanaka Y, Emoto K, Cai Z, Aoki T, Schlichting D, Rooney T, Macias W: Efficacy and Safety of Baricitinib in Japanese Patients with Active Rheumatoid Arthritis Receiving Background Methotrexate Therapy: a 12-week, Double-blind, Randomized Placebo-controlled Study. Journal of rheumatology 2016, 43(3):504‐511.

23. Tanaka Y, Ishii T, Cai Z, Schlichting D, Rooney T, Macias W: Efficacy and safety of baricitinib in Japanese patients with active rheumatoid arthritis: a 52-week, randomized, single-blind, extension study. Modern rheumatology 2018, 28(1):20‐29.

24. Taylor PC, Keystone EC, van der Heijde D, Weinblatt ME, Del Carmen Morales L, Reyes Gonzaga J, Yakushin S, Ishii T, Emoto K, Beattie S et al: Baricitinib versus Placebo or Adalimumab in Rheumatoid Arthritis. The New England journal of medicine 2017, 376(7):652-662.

25. Combe B, Kivitz A, Tanaka Y, van der Heijde D, Simon JA, Baraf HSB, Kumar U, Matzkies F, Bartok B, Ye L et al: Filgotinib versus placebo or adalimumab in patients with rheumatoid arthritis and inadequate response to methotrexate: a phase III randomised clinical trial. Annals of the rheumatic diseases 2021, 80(7):848-858.

26. Genovese MC, Kalunian K, Gottenberg JE, Mozaffarian N, Bartok B, Matzkies F, Gao J, Guo Y, Tasset C, Sundy JS et al: Effect of Filgotinib vs Placebo on Clinical Response in Patients With Moderate to Severe Rheumatoid Arthritis Refractory to Disease-Modifying Antirheumatic Drug Therapy: The FINCH 2 Randomized Clinical Trial. Jama 2019, 322(4):315-325.

27. Kavanaugh A, Kremer J, Ponce L, Cseuz R, Reshetko OV, Stanislavchuk M, Greenwald M, Van der Aa A, Vanhoutte F, Tasset C et al: Filgotinib (GLPG0634/GS-6034), an oral selective JAK1 inhibitor, is effective as monotherapy in patients with active rheumatoid arthritis: results from a randomised, dose-finding study (DARWIN 2). Annals of the rheumatic diseases 2017, 76(6):1009-1019.

28. Westhovens R, Taylor PC, Alten R, Pavlova D, Enríquez-Sosa F, Mazur M, Greenwald M, Van der Aa A, Vanhoutte F, Tasset C et al: Filgotinib (GLPG0634/GS-6034), an oral JAK1 selective inhibitor, is effective in combination with methotrexate (MTX) in patients with active rheumatoid arthritis and insufficient response to MTX: results from a randomised, dose-finding study (DARWIN 1). Annals of the rheumatic diseases 2017, 76(6):998-1008.

29. Genovese MC, Greenwald M, Codding C, Zubrzycka-Sienkiewicz A, Kivitz AJ, Wang A, Shay K, Wang X, Garg JP, Cardiel MH: Peficitinib, a JAK Inhibitor, in Combination With Limited Conventional Synthetic Disease-Modifying Antirheumatic Drugs in the Treatment of Moderate-to-Severe Rheumatoid Arthritis. Arthritis & rheumatology (Hoboken, NJ) 2017, 69(5):932‐942.

30. Genovese MC, Greenwald MW, Gutierrez-Ureña SR, Cardiel MH, Poiley JE, Zubrzycka-Sienkiewicz A, Codding CE, Wang A, He W, Amos R et al: Two-Year Safety and Effectiveness of Peficitinib in Moderate-To-Severe Rheumatoid Arthritis: A Phase IIb, Open-Label Extension Study. Rheumatology and therapy 2019, 6(4):503-520.

31. Kivitz AJ, Gutierrez-Ureña SR, Poiley J, Genovese MC, Kristy R, Shay K, Wang X, Garg JP, Zubrzycka-Sienkiewicz A: Peficitinib, a JAK Inhibitor, in the Treatment of Moderate-to-Severe Rheumatoid Arthritis in Patients With an Inadequate Response to Methotrexate. Arthritis & rheumatology (Hoboken, NJ) 2017, 69(4):709‐719.

32. Takeuchi T, Tanaka Y, Iwasaki M, Ishikura H, Saeki S, Kaneko Y: Efficacy and safety of the oral Janus kinase inhibitor peficitinib (ASP015K) monotherapy in patients with moderate to severe rheumatoid arthritis in Japan: a 12-week, randomised, double-blind, placebo-controlled phase IIb study. Annals of the rheumatic diseases 2016, 75(6):1057‐1064.

33. Takeuchi T, Tanaka Y, Tanaka S, Kawakami A, Iwasaki M, Katayama K, Rokuda M, Izutsu H, Ushijima S, Kaneko Y et al: Efficacy and safety of peficitinib (ASP015K) in patients with rheumatoid arthritis and an inadequate response to methotrexate: results of a phase III randomised, double-blind, placebo-controlled trial (RAJ4) in Japan. Annals of the rheumatic diseases 2019, 78(10):1305-1319.

34. Takeuchi T, Tanaka Y, Tanaka S, Kawakami A, Song YW, Chen YH, Rokuda M, Izutsu H, Ushijima S, Kaneko Y et al: Safety and effectiveness of peficitinib (ASP015K) in patients with rheumatoid arthritis: interim data (22.7 months mean peficitinib treatment) from a long-term, open-label extension study in Japan, Korea, and Taiwan. Arthritis research & therapy 2020, 22(1).

35. Takeuchi T, Tanaka Y, Tanaka S, Kawakami A, Song YW, Chen YH, Rokuda M, Izutsu H, Ushijima S, Kaneko Y: Safety and Effectiveness of Peficitinib (ASP015K) in Patients with Rheumatoid Arthritis: Final Results (32 Months of Mean Peficitinib Treatment) From a Long-Term, Open-Label Extension Study in Japan, Korea, and Taiwan. Rheumatology and therapy 2021, 8(1):425-442.

36. Tanaka Y, Takeuchi T, Tanaka S, Kawakami A, Iwasaki M, Song YW, Chen YH, Wei JC, Lee SH, Rokuda M et al: Efficacy and safety of peficitinib (ASP015K) in patients with rheumatoid arthritis and an inadequate response to conventional DMARDs: a randomised, double-blind, placebo-controlled phase III trial (RAJ3). Annals of the rheumatic diseases 2019, 78(10):1320-1332.

37. Burmester GR, Kremer JM, Van den Bosch F, Kivitz A, Bessette L, Li Y, Zhou Y, Othman AA, Pangan AL, Camp HS: Safety and efficacy of upadacitinib in patients with rheumatoid arthritis and inadequate response to conventional synthetic disease-modifying anti-rheumatic drugs (SELECT-NEXT): a randomised, double-blind, placebo-controlled phase 3 trial. Lancet (London, England) 2018, 391(10139):2503‐2512.

38. Fleischmann R, Pangan AL, Song IH, Mysler E, Bessette L, Peterfy C, Durez P, Ostor AJ, Li Y, Zhou Y et al: Upadacitinib Versus Placebo or Adalimumab in Patients With Rheumatoid Arthritis and an Inadequate Response to Methotrexate: Results of a Phase III, Double-Blind, Randomized Controlled Trial. Arthritis & rheumatology (Hoboken, NJ) 2019, 71(11):1788-1800.

39. Fleischmann RM, Genovese MC, Enejosa JV, Mysler E, Bessette L, Peterfy C, Durez P, Ostor A, Li Y, Song IH: Safety and effectiveness of upadacitinib or adalimumab plus methotrexate in patients with rheumatoid arthritis over 48 weeks with switch to alternate therapy in patients with insufficient response. Annals of the rheumatic diseases 2019, 78(11):1454‐1462.

40. Genovese MC, Smolen JS, Weinblatt ME, Burmester GR, Meerwein S, Camp HS, Wang L, Othman AA, Khan N, Pangan AL et al: Efficacy and Safety of ABT-494, a Selective JAK-1 Inhibitor, in a Phase IIb Study in Patients With Rheumatoid Arthritis and an Inadequate Response to Methotrexate. Arthritis & rheumatology (Hoboken, NJ) 2016, 68(12):2857-2866.

41. Genovese MC, Fleischmann R, Combe B, Hall S, Rubbert-Roth A, Zhang Y, Zhou Y, Mohamed MF, Meerwein S, Pangan AL: Safety and efficacy of upadacitinib in patients with active rheumatoid arthritis refractory to biologic disease-modifying anti-rheumatic drugs (SELECT-BEYOND): a double-blind, randomised controlled phase 3 trial. Lancet (London, England) 2018, 391(10139):2513‐2524.

42. Kameda H, Takeuchi T, Yamaoka K, Oribe M, Kawano M, Zhou Y, Othman AA, Pangan AL, Kitamura S, Meerwein S et al: Efficacy and safety of upadacitinib in Japanese patients with rheumatoid arthritis (SELECT-SUNRISE): a placebo-controlled phase IIb/III study. Rheumatology (Oxford, England) 2020, 59(11):3303-3313.

43. Kameda H, Takeuchi T, Yamaoka K, Oribe M, Kawano M, Yokoyama M, Pangan AL, Konishi Y, Meerwein S, Tanaka Y: Efficacy and safety of upadacitinib over 84 weeks in Japanese patients with rheumatoid arthritis (SELECT-SUNRISE). Arthritis research & therapy 2021, 23(1):9.

44. Kremer JM, Emery P, Camp HS, Friedman A, Wang L, Othman AA, Khan N, Pangan AL, Jungerwirth S, Keystone EC: A Phase IIb Study of ABT-494, a Selective JAK-1 Inhibitor, in Patients With Rheumatoid Arthritis and an Inadequate Response to Anti-Tumor Necrosis Factor Therapy. Arthritis and rheumatology 2016, 68(12):2867‐2877.

45. Rubbert-Roth A, Enejosa J, Pangan AL, Haraoui B, Rischmueller M, Khan N, Zhang Y, Martin N, Xavier RM: Trial of Upadacitinib or Abatacept in Rheumatoid Arthritis. New England journal of medicine 2020, 383(16):1511‐1521.

46. Smolen JS, Pangan AL, Emery P, Rigby W, Tanaka Y, Vargas JI, Zhang Y, Damjanov N, Friedman A, Othman AA et al: Upadacitinib as monotherapy in patients with active rheumatoid arthritis and inadequate response to methotrexate (SELECT-MONOTHERAPY): a randomised, placebo-controlled, double-blind phase 3 study. Lancet (London, England) 2019, 393(10188):2303‐2311.

47. Vollenhoven R, Takeuchi T, Pangan AL, Friedman A, Mohamed MF, Chen S, Rischmueller M, Blanco R, Xavier RM, Strand V: Efficacy and Safety of Upadacitinib Monotherapy in Methotrexate‐Naive Patients With Moderately‐to‐Severely Active Rheumatoid Arthritis (SELECT‐EARLY): a Multicenter, Multi‐Country, Randomized, Double‐Blind, Active Comparator–Controlled Trial. Arthritis & rheumatology 2020, 72(10):1607‐1620.
